# Convection-Enhanced Delivery of AAV2-PrPshRNA in Prion-Infected Mice

**DOI:** 10.1371/journal.pone.0098496

**Published:** 2014-05-27

**Authors:** Misol Ahn, Krystyna Bajsarowicz, Abby Oehler, Azucena Lemus, Krystof Bankiewicz, Stephen J. DeArmond

**Affiliations:** 1 Department of Pathology, University of California San Francisco, San Francisco, California, United States of America; 2 Department of Neurosurgery and Neurology, University of California San Francisco, San Francisco, California, United States of America; 3 Department of Institute for Neurodegenerative Diseases, University of California San Francisco, San Francisco, California, United States of America; Ruhr University Bochum, Germany

## Abstract

Prion disease is caused by a single pathogenic protein (PrP^Sc^), an abnormal conformer of the normal cellular prion protein PrP^C^. Depletion of PrP^C^ in prion knockout mice makes them resistant to prion disease. Thus, gene silencing of the *Prnp* gene is a promising effective therapeutic approach. Here, we examined adeno-associated virus vector type 2 encoding a short hairpin RNA targeting *Prnp* mRNA (AAV2-PrP-shRNA) to suppress PrP^C^ expression both *in vitro* and *in vivo*. AAV2-PrP-shRNA treatment suppressed PrP levels and prevented dendritic degeneration in RML-infected brain aggregate cultures. Infusion of AAV2-PrP-shRNA-eGFP into the thalamus of CD-1 mice showed that eGFP was transported to the cerebral cortex via anterograde transport and the overall PrP^C^ levels were reduced by ∼70% within 4 weeks. For therapeutic purposes, we treated RML-infected CD-1 mice with AAV2-PrP-shRNA beginning at 50 days post inoculation. Although AAV2-PrP-shRNA focally suppressed PrP^Sc^ formation in the thalamic infusion site by ∼75%, it did not suppress PrP^Sc^ formation efficiently in other regions of the brain. Survival of mice was not extended compared to the untreated controls. Global suppression of PrP^C^ in the brain is required for successful therapy of prion diseases.

## Introduction

Prion diseases such as Creutzfeldt Jacob disease (CJD), Gerstmann-Sträussler-Scheinker disease, kuru and variant CJD caused by bovine spongiform encephalopathy are some of the most devastating neurodegenerative diseases [1]. Abnormal conformational changes in the normal cellular prion protein (PrP^C^) produce the pathogenic forms of prion protein (PrP^Sc^). Accumulation of PrP^Sc^ in neuronal membranes, autosomes and lysosomes results in synapse degeneration within a few weeks and nerve cell loss at the later stage of the disease [2]–[4].

Developing drug treatments for prion diseases presents significant challenges. Drugs must cross the blood brain barrier (BBB) to reach to the central nervous system (CNS) efficiently and the treatment must clear all strains of PrP^Sc^. Quinacrine (Qa) is a successful anti-prion compound *in vitro*; however, it does not cross the BBB efficiently and Qa failed to treat prion disease *in vivo* [5]–[10]. Qa treatment of MDR1^0/0^ mice in which the BBB is impaired showed significantly increased bioavailability in the brain [11], but Qa treatment of MDR1^0/0^ mice infected with the Rocky Mountain Laboratory prion strain (RML) starting at 60 days post inoculation (60 dpi) cleared PrP^Sc^ in the brain only for the first 15 days of treatment but Qa-resistant strains of PrP^Sc^ rebounded rapidly and mice died of prion disease[12]. Although abnormal conformers of PrP^C^ are designated as the single term of PrP^Sc^, there are many different conformers of PrP^Sc^ that comprise a prion strain [13]. This may be why administration of single drugs midway during the prion incubation period has not improved survival more than 20–30% [14]–[17]. Dual drug treatment with Qa and the γ-secretase inhibitor (GSI), LY411575, in RML-infected CD-1 mice showed that dendritic degeneration was prevented and PrP^Sc^ formation was halted for up to 7 weeks once the treatment started at 50 dpi [10]. However, attempts at longer-term continuous treatment of Qa and GSI failed due to GSI toxicity. Shorter 28-day periods of Qa and GSI treatment starting at 50 dpi showed rebound of PrP^Sc^ 3 weeks after the end of treatment and the mice died of prion diseases. These studies suggest the limitations of current drug therapy and the need of new approaches.

Another approach to treat prion disease is to prevent formation of PrP^Sc^ by depleting PrP^C^. Knocking out the *Prnp* gene in mice prevented prion disease without producing significantly disabling defects [18]. Therefore, introducing a short-hairpin RNA (shRNA) in adult mice to suppress PrP^C^ expression appears to be a plausible strategy to prevent prion disease. Convection enhanced delivery (CED) of adeno-associated virus (AAV) vectors has been shown to deliver gene products efficiently throughout the brain in non-human primates [19]–[21]. Unlike diffusion-based delivery, CED uses bulk flow or fluid convection as a result of a pressure gradient rather than a concentration gradient [22]. With a pressure gradient from the delivery cannula tip, CED is able to deliver small and large molecules to clinically significant target volumes, centimeters rather than millimeters in diameter [22], [23]. The AAV type 2 vector (AAV2) has been widely used to treat brain tumors and Parkinson's disease without toxicity [24]–[28]. We hypothesized that gene therapy to suppress PrP^C^ expression in combination with Qa and GSI drug treatment might be effective. Here, we delivered AAV2-PrP-shRNA to suppress PrP^C^ expression both *in vitro* and *in vivo*. Introducing AAV2-PrP-shRNA significantly reduced total PrP in RML-infected brain aggregates (BrnAggs) and produced ∼70% suppression of PrP^C^ expression in the brain of uninfected CD-1 mice. However, we observed only focal suppression of PrP^Sc^ in RML-infected mice treated with AAV2-PrP-shRNA and mice did not show improved survival. This study suggests that this gene construct will not produce the global suppression of PrP^C^ throughout the brain required for effective treatment for prion diseases.

## Results

### AAV2-PrP-shRNA significantly reduces PrP^C^ synthesis and PrP^Sc^ formation *in vitro*

**Figure 1:**
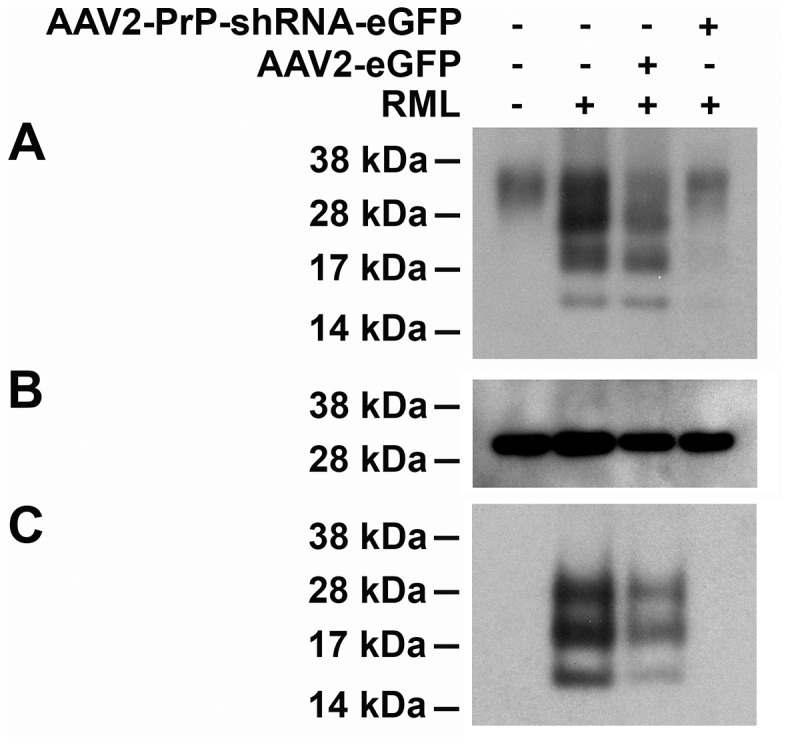
AAV2-PrP-shRNA-eGFP treatment reduced PrP^Sc^ in RML-infected BrnAggs. BrnAggs were treated with AAV2-eGFP or AAV2-PrP-shRNA-eGFP 4 days before RML infection at day 17 in culture and harvested at 41 days in culture. Uninfected and RML-infected/untreated controls were also collected at 41 days in culture. 12 brain aggregates were pooled together in duplicates. **A**. 5 µg of protein lysate was loaded in each lane of a SDS-PAGE gel. The total PrP in each sample was analyzed by Western blot analysis. **B**. The blot of **A** was stripped and reblotted with an anti-actin antibody. **C**. 25 µg of protein lysate were digested with PK at 37°C for 1 hr, run in a SDS-PAGE gel and immunoblotted with a D13-HRP antibody for PrP^Sc^ detection.

First, we studied the effect of AAV2-PrP-shRNA and AAV2-eGFP (enhanced green fluorescent protein) on PrP^C^ and PrP^Sc^ expression in BrnAggs *in vitro*. BrnAggs contain mature neurons, astrocytes, oligodendrocytes and microglia [29]. We exposed BrnAggs 13 days in culture (dic) to either AAV2-eGFP or to AAV2-PrP-shRNA-eGFP (both at 2×10^9^ viral genomes per ml) followed by infection with RML at 17 dic. Four groups of BrnAggs were studied in duplicate: uninfected, RML-infected, RML-infected/treated with AAV2-eGFP and RML-infected/treated with AAV2-PrP-shRNA-eGFP (**Fig. 1**). BrnAggs were harvested at 41 dic. For Western analysis, a pool of 12 BrnAggs were prepared for each sample. 5 µg of protein lysate from each sample were loaded into a SDS-PAGE gel to measure total PrP (PrP^C^ and PrP^Sc^) expression. We observed ∼80% reduction of total PrP in BrnAggs treated with AAV2-PrP-shRNA-eGFP compared to untreated or AAV2-eGFP treated controls (**Fig. 1A**). In order to measure the level of PrP^Sc^, 25 µg of protein lysate from each sample were digested with protease K (PK). The levels of PrP^C^ and PrP^Sc^ were significantly reduced with AAV2-PrP-shRNA-eGFP treatment (**Fig. 1A and 1C**). Protein loads were tested with actin (**Fig. 1B**). We observed that significant reduction in PrP^Sc^ with AAV2-PrP-shRNA-eGFP treatment in RML-infected BrnAggs.

### Dendritic degeneration in RML-infected BrnAggs is prevented by AAV2-PrP-shRNA treatment

**Figure 2:**
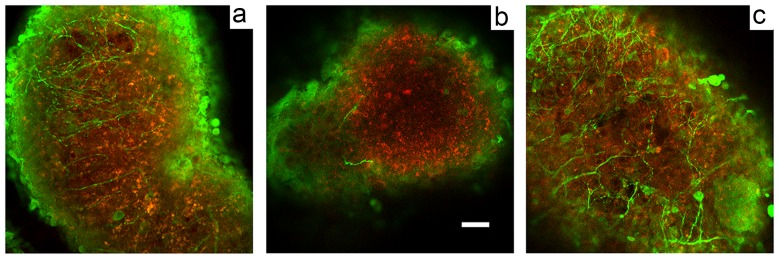
Dendritic degeneration in RML-infected BrnAggs was prevented with AAV2-PrP-shRNA treatment. 3 groups of BrnAggs were analysed: uninfected (**a**), infected with RML at 15 days in culture (**b**) and treated with AAV2-PrP-shRNA-eGFP for 10 days after RML infection (**d**). BrnAggs were stained with anti-MAP2 (green, dendrites) and anti-PrP (red, prion protein). Bar  = 50 µm; n = 3.

Previously we showed that RML infection induces progressive dendritic degeneration in BrnAggs [29]. We observed significant dendritic degeneration in RML-infected BrnAggs from 10 days post infection (dpi) when compared to age-matched uninfected controls. In order to evaluate whether AAV2-PrP-shRNA treatment can prevent dendritic degeneration, we examined dendrite density of uninfected, RML-infected and RML-infected/AAV2-PrP-shRNA-treated BrnAggs after 20 dpi. BrnAggs fixed in 4% formaldehyde were stained with an anti-MAP2 and D18 antibodies for dendritic tree and prion protein, respectively. Confocal 1- µm thick images in stacks of 12–15 were collected from each sample. Samples are shown in **Fig. 2**. Dendritic loads of AAV2-PrP-shRNA-treated BrnAggs (**Fig. 2c**) are very similar to uninfected BrnAggs (**Fig. 2a**); in contrast, most dendrites were degenerated in untreated RML-infected BrnAgg (**Fig. 2b**).

### AAV2-PrP-shRNA suppresses PrP^C^ expression in the brains of wild-type CD-1 mice

Uninfected CD-1 mice (n = 4) were infused intrathalamically with AAV2-PrP-shRNA-eGFP by stereotaxic convection-enhanced delivery (CED) in order to determine the number of brain regions transduced by the AAV2 vector and to test whether infusion of AAV2-PrP-shRNA suppresses PrP^C^ expression in the brain. Four µl of AAV2-PrP-shRNA-eGFP (1.07×10^13^ vg/ml) were infused bilaterally into each side of the thalamus at a rate of 0.5 µl/m in uninfected wild-type CD-1 mice (total of 8 µl) with the two cannulas separated by 2 mm. Mice infused with AAV2-PrP-shRNA-eGFP did not show any adverse effects after the infusion. After 30 days, the mice were sacrificed and brains were either perfused with 4% paraformaldehyde for immunohistochemical staining with an anti-GFP antibody or flash-frozen to examine the level of PrP expression with histoblot analysis.

**Figure 3:**
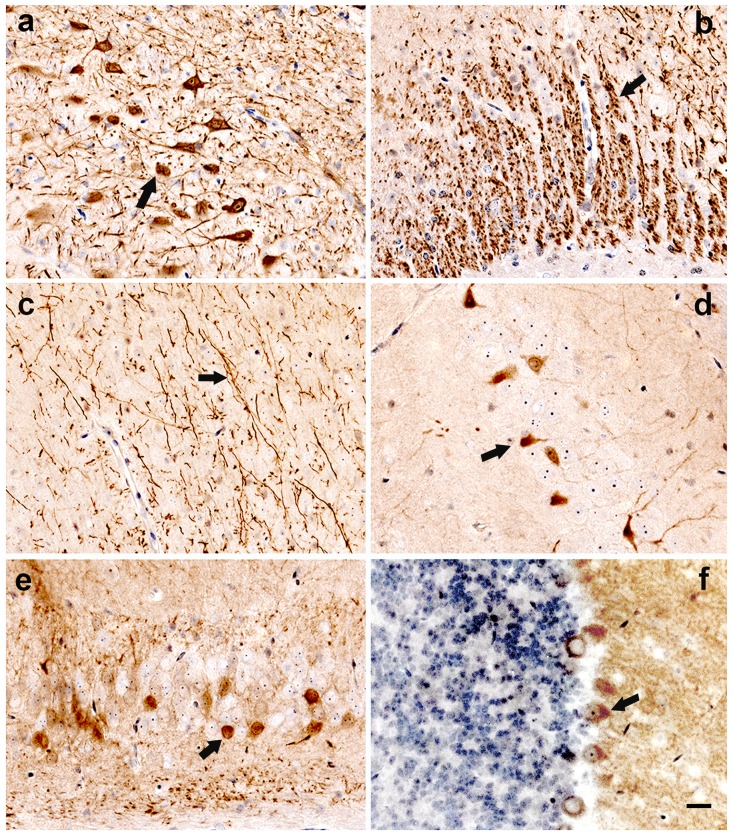
eGFP expression of CD-1 mice infused with AAV2-PrP-shRNA-eGFP. CD-1 mice (n = 4) were infused with 4 µl of AAV2-PrP-shRNA-eGFP bilaterally into the thalamus. Collected brains were flash-frozen 30 days after infusion. Immunohistochemical staining of eGFP showed that eGFP was transported from the dorsal thalamus to the hippocampus and cortex. (**a**) Dorsal thalamus. (**b**) Cingulum bundle (arrow). (**c**) Layer 6 of cerebral cortex. (**d**) CA1 region of hippocampus (arrow). (**e**) Dentate gyrus (arrow). (**f**) Cerebellar cortex; Purkinje cells (arrow). Bar  = 50 µm (**a–e**).

The highest expression of eGFP was seen in neurons of the dorsal thalamus, where AAV2-eGFP was infused (**Fig. 3a**). eGFP was also observed in the axonal projections from the dorsal thalamus to the cingulum bundle associated with internal capsule (**Fig. 3b**), in layer 6 of the neocortex (**Fig. 3c**), and in the entorhinal cortex. eGFP expression was found in the thalamic axonal projections to the cortex but not in neuronal cell bodies of the cerebral cortex (**Fig. 3c**), suggesting that AAV2 transported PrP-shRNA via anterograde axonal transport in mice but it failed to cross synapses to transduce cortical neurons. eGFP was also expressed in neurons of the CA1 region of the hippocampus (**Fig. 3d**), the dentate gyrus of the hippocampus (**Fig. 3e**) and the Purkinje cell layer in the cerebellum (**Fig. 3f**).

**Figure 4:**
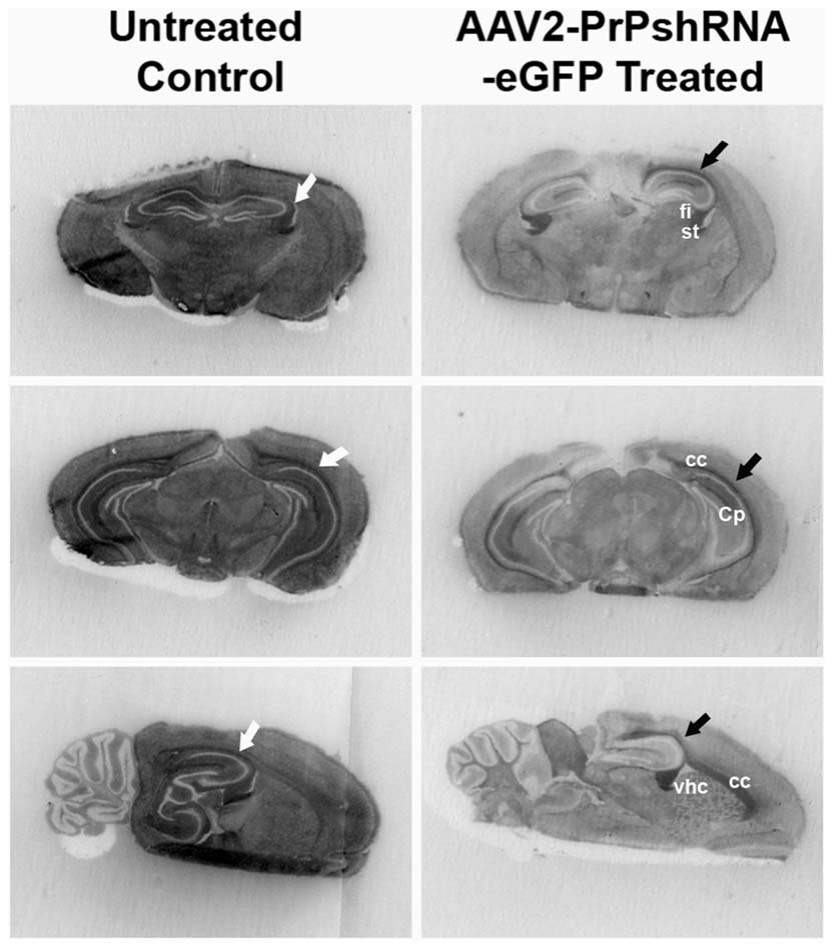
PrP^C^ expression of CD-1 mice infused with AAV2-PrP-shRNA-eGFP. CD-1 mice (n = 4) were infused with 4 ul of AAV2-shRNA-PrP-eGFP bilaterally into the thalamus and the brain was collected flash-frozen 30 days after infusion. The coronal sections of cerebellum/brainstem (Cb/Bs), midbrain (Mb) and hippocampus/thalamus (Hp) or sagittal sections of the brain were transferred into nitrocellulose paper and stained with D18 antibody for PrP^C^ detection. fi (fimbria hippocampus), st (stria terminals), cc (corpus callosum), Cp (caudate putamen), vhc (vent hip commissure). Arrow (corpus callosum).

We also examined whether PrP^C^ expression was suppressed in the AAV2-PrP-shRNA-eGFP treated brains of uninfected CD-1 mice with histoblot analysis (**Fig. 4**). The intergraded optical densitometry measurements of PrP^C^ signals from the histoblots of untreated and AAV2-PrP-shRNA-eGFP treated mice were determined by BioQuant morphometry and we found that expression of PrP^C^ was reduced about 74% in the brains treated with AAV2-PrP-shRNA-eGFP compared to the brains of untreated mice; however, intense staining of PrP^C^ was still observed in the white matter tracts of the corpus callosum, fimbria hippocampus and ventral hippocampal commissure (**Fig. 4**).

### AAV2-PrP-shRNA treatment did not extend the survival of prion-infected CD-1 mice

Previously we reported that 50 days of combined GSI and Qa treatment, begun at 50 dpi, significantly decreased PrP^Sc^ in the gray matter of thalamus, hippocampus and cerebral cortex, but allowed PrP^Sc^ to be stored in the white matter [10]. A short course of the Qa and GSI combination could provide a temporary halt to PrP^Sc^ formation while the gene therapy construct might spread well enough to prevent storage of PrP^Sc^ in the white matter tracts thus preventing rebound of the disease when drug therapy was stopped and extending the survival time or RML-infected mice.

We speculated that the infusion of AAV2-PrP-shRNA given in combination with GSI+Qa treatment might prevent PrP^Sc^ formation. RML-infected CD-1 mice were treated with AAV2-PrP-shRNA at 50 dpi with or without 28 days of concomitant oral treatment of GSI+Qa. However, the combination therapy did not extend survival. Dual treatment of gene- and pharmaco-therapy gave the mean survival of 131 dpi (a range from 114 to 146 dpi, n = 12), which was similar to the control untreated group with a mean survival of 136 dpi (a range from 108 to 146 dpi, n = 15) (**Table 1**).

**Table 1:** Survival of RML-infected CD-1 mice treated with AAV2-PrP-shRNA and GSI/Qa was very similar to that of untreated control mice.

	Untreated Control	Treated with AAV2-PrPshRNA	Treated with AAV2-PrPshRNA + Qa/GSI
Days post inoculation when each individual mouse diagnosed with prion disease	108	90	114
	127	90	115
	133	94	120
	136	104	120
	136	115	122
	136	115	136
	136	115	136
	136	136	136
	136	140	140
	140	140	146
	146	146	146
	146	155	146
	146		
	146		
	146		
Average survival time (Days)	136.9±9.9	120±22.9	131.4±12.4
Number of sick mice/number of mice per group	15/15	12/12	12/12

4 µl of AAV2-PrP-shRNA was infused to the thalamus bilaterally at 50 dpi and the next day, AAV-infused mice were treated with 5 mg/kg/day of GSI and 40 mg/kg/day of Qa for 28 days. The average survival time was calculated based on the onset of prion disease. Mice were euthanized one day after they showed scrapie-sick symptoms.

**Figure 5:**
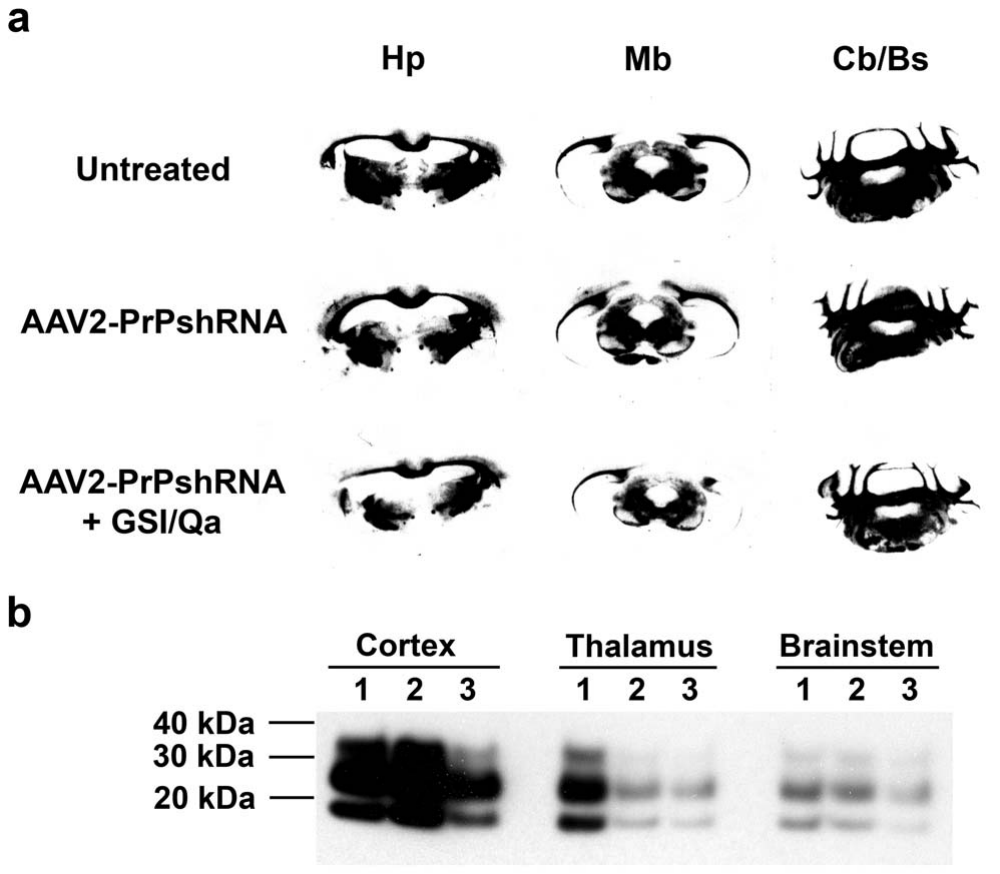
The level of PrP^Sc^ was reduced only in the grey matter of the thalamus of RML-infected CD-1 mice treated with AAV2-PrP-shRNA. **a**. CD-1 mice were inoculated with RML and i) untreated (n = 15), ii) infused with AAV2-PrP-shRNA at 50 dpi (n = 12) or iii) infused with AAV2-PrP-shRNA in combination with GSI/Qa treatment for 28 days at 50 dpi (n = 12). Both inoculation and infusion was into the thalamus. Scrapie-sick mice were euthanized and the brain samples were collected for flash-frozen, sectioned (10 µm thickness) and analyzed by histoblot with PK digestion. Samples shown in the figure are from mice of each group died of prion disease around 140 dpi. **b**. The level of PrP^Sc^ was measured by Western blot analysis. Flash frozen brains were micro-dissected into three regions; the cortex, thalamus and brainstem. 5 µg of protein lysates were digested with PK and run in a SDS PAGE gel (1: untreated control, 2: RML-infected mice treated with AAV2-PrP-shRNA at 50 dpi, 3: RML-infected mice treated with AAV2-PrP-shRNA with a combination of GSI and Qa treatment for 28 days of oral administration at 50 dpi). Samples shown in the figure are mice of each group died of prion disease around 118–136 dpi.

**Figure 6:**
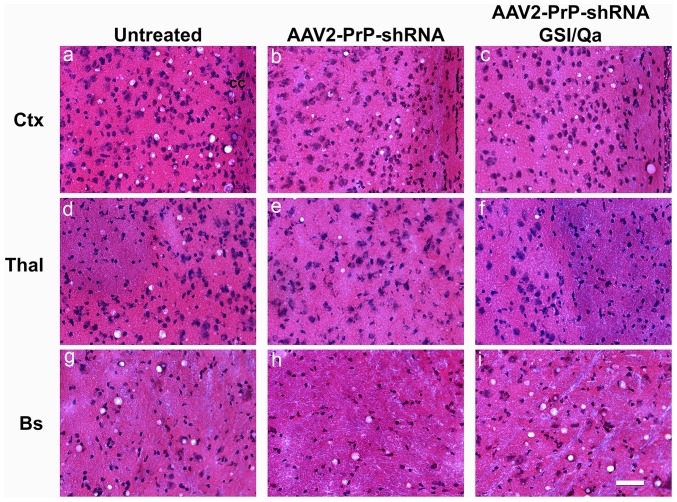
Vacuolation in the thalamus, where AAV2-PrP-shRNA was infused, was reduced in RML-infected mice treated with AAV2-PrP-shRNA. Flash frozen brain sections (10 µm) were used for H&E staining. Vacuolation was reduced in AAV2-PrP-shRNA treated mice by about 50% compared to untreated control mice. RML-infected mice treated with both AAV2-PrP-shRNA and drug combination (GSI and Qa) showed more vacuolation than the control in the brainstem. Ctx (cortex), Thal (thalamus), Bs (brainstem). A bar  = 50 µm.

With Histoblot analysis we examined PrP^Sc^ in the brain of RML-infected CD-1 mice treated with AAV2-PrP-shRNA. Brain sections of untreated, AAV2-PrPshRNA treated and AAV2-PrPshRNA/GSI/Qa treated mice were transferred into the nitrocellulose membrane, digested with PK and stained for PrP^Sc^ detection. We found only a mild but definite reduction of PrP^Sc^ in the dorsal thalamus but not in the ventral thalamus containing the majority of white matter tracks (**Fig. 5a**). Other white matter tracts containing large amounts of PrP^Sc^ were the corpus callosum and the deep white matter of the cerebellum. Overall, PrP^Sc^ levels in the cortex and brainstem were very similar or slightly lower in treated groups; however, PrP^Sc^ in the thalamus was much reduced (**Fig. 5b**). Vacuolation in the thalamus of AAV2-PrP-shRNA treated mice (**Fig. 6e**) was slightly lower compared to the untreated controls (**Fig. 6d**) but vacuolation in other brain regions were indistinguishable between treated and untreated groups (**Fig. 6** **, a–c and g–i**).

## Discussion

Gene therapy for prion disease appears very promising because prion disease is caused by a single protein, namely the conversion of normal cellular PrP^C^ to pathogenic PrP^Sc^. Without PrP^C^, PrP^Sc^ cannot be formed. Unlike many other proteins, knocking down PrP^C^ in mice does not show detrimental effects during development or postnatally [18], [30]. While homozygous Prnp^o/o^ knockout mice are completely resistant to prion disease, heterozygous Prnp^o/+^ knockdown mice that have 50% less PrP^C^ show significantly enhanced resistance to prion infection with about a doubling of survival time compared to the controls [18], [31]. Depletion of neuronal PrP^C^ in transgenic mice after prion infection can also prevent prion disease [32]. RNAi studies with a lentiviral vector carrying PrP-shRNA reported prolonged survival of prion-infected mice [33], [34]; however, the greatest effect was observed when PrP^C^ expression was suppressed prior to prion infection [34]. When a lentiviral vector carrying a PrP-shRNA was infused bilaterally in the hippocampus of mice at 63 dpi, survival time was extended by 23.5% (range of survival, 89–129 dpi) [33]. We adapted their PrP-shRNA sequence in our studies.

This study was performed with an AAV2 viral vector carrying a PrP-shRNA in an attempt to prevent PrP^C^ synthesis and thus to prevent PrP^Sc^ formation. We believed AAV2 would be a satisfactory vector to deliver a PrP-shRNA because it is known to deliver its gene product in neurons and it has shown to be effective in treating Parkinsonism [27], [35]. In our preliminary *in vitro* studies, AAV2-PrP-shRNA treatment of RML-infected BrnAggs significantly reduced PrP^Sc^ levels and prevented dendritic degeneration. In addition, we observed about 74% decreases in PrP^C^ in the brains of uninfected CD-1 mice treated with AAV2-PrP-shRNA (**Fig. 4**). In spite of these encouraging results, there was no extension of survival in RML-infected CD-1 mice following AAV2-PrP-shRNA treatment beginning at 50 dpi (**Table 1**). The pathogenic processes set in motion by prion infection before treatment was begun could not be completely corrected by the AAV2-PrP-shRNA because it did not cross synapses in the cortex, brainstem and other brain regions interconnected with the thalamus (the site of infusion of AAV2-PrP-shRNA) even though the AAV2-PrP-shRNA was carried to the cerebral cortex and other brain regions by axonal transport.

Combining 28 days of GSI and Qa treatment with AAV2-PrP-shRNA also resulted in no survival extension although higher reduction of PrP^Sc^ levels in the thalamus and cerebral cortex was observed (**Fig. 5b**). From histoblot data, we found that AAV2-PrP-shRNA treatment in both uninfected and RML-infected CD-1 mice did not remove PrP^C^ from the white matter (**Fig. 4** and **Fig. 5a**) and hence did not prevent PrP^Sc^ formation completely. The presence of PrP^Sc^ in white matter tracts may block the axonal transport of essential cytoplasmic proteins to synapses; these proteins are necessary to maintain the viability of post-synaptic neurons. In addition, accumulation of PrP^Sc^ in synaptic terminals leads to synapse degeneration and subsequent accumulation of PrP^Sc^ in post-synaptic neuronal cell membranes which causes dendritic degeneration by a Notch-1 activation mechanism [4], [10].

In summary treatment with AAV2-PrP-shRNA decreased PrP^Sc^ in the thalamic gray matter by more than 95% but had no effect on brainstem PrP^Sc^. Treatment with AAV2-PrP-shRNA in combination with GSI+Qa decreased PrP^Sc^ in the cerebral cortex by 75%, in the thalamus by greater than 95%, and in the brainstem by greater than 75% (**Fig. 5b**) but long-term combined treatment was not possible because of toxicity associated with GSI. These results also suggest that the mouse may not be an appropriate surrogate model system for human prion disease because, in human CJD, PrP^Sc^ does not accumulate in the white matter but only in the gray matter [36].

Currently, we are testing other AAV serotypes such as AAV1, AAV6 and AAV9, which seem to be delivered to broader regions of the brain via retrograde axonal transport [37]–[40]. Retrograde transport ensures that axons projecting into a brain region where the gene therapy is delivered will transport the construct back to the nerve cell bodies and transduce neurons in distant brain regions. We are also testing different infusion sites of gene delivery. In the lentivirus study noted above, mice were inoculated with RML into the right parietal lobe, not into the thalamus. It will be interesting to examine whether inoculation sites affect the rate of disease progression or patterns of prion disease. Although our *in vivo* study did not lead to extension of survival of prion-infected mice, we showed that suppression of PrP^C^ expression could reduce formation of PrP^Sc^ and dendritic degeneration both *in vitro* and *in vivo*. This study confirms that, in order to be a successful therapy for prion disease, it is critical to deliver PrP-shRNA efficiently and globally throughout the brain. These initial results with gene and drug therapy are promising enough to encourage further investigation of combined therapy for prion disease.

## Materials and Methods

All experiments were carried out in accordance with the Institutional Animal Care and Use Committee/Laboratory Animal Research Center (IACUC) protocol of the University of California, San Francisco (UCSF). The protocol was approved by the UCSF IACUC Committee (Authorization protocol number: AB091167–02). All surgery was performed under anesthesia and all efforts were made to minimize suffering. Mice are euthanized with isofluorane overdose followed by cervical dislocation.

### Animals

E15-days gestation FVB embryos were used to prepare BrnAggs. Approximately five-week-old female wild-type (CD-1) mice for survival experiment were purchased from Charles River (Wilmington, MA).

### Materials

Quinacrine dihydrochloride (Qa) (69–05–6, Sigma-Aldrich, St. Louise, MO), LY411575 (GSI) (a kindly gift by Dr. Golde, Mayo Clinic, Jacksonville, FL), neuroblastoma cells expressing 6× PrP^C^, N2a.cl3, (a kindly gift from Dr. Ghaemmaghami, UCSF, San Francisco, CA)[41], gentamycin, 50 mg/ml, penicillin-streptomycin, 10,000 units/ml and 10,000 µg/ml, respectively, GlutaMAX, Dithiothreitol (DTT) and proteinase K (PK), Hoechst (Invitrogen), Complete protease inhibitor cocktail tablets (Roche, Indianapolis), Western blotting detection reagents (GE Healthcare), anti-MAP2 antibody (AB5622, Millipore), anti-GFAP (20334, Dako), anti-actin (MA515739, Thermo Scientific), anti-PrP antibodies, R2, D18, D13, HumP, and D13 conjugated with horseradish peroxidase (HRP) (from Dr. Prusiner, UCSF, San Francisco, CA), Donkey anti-rabbit IgG conjugated with Alexa488 and Donkey anti-human IgG conjugated with Alexa594 (Jackson Immunoresearch Laboratory) and AAV2 constructs (Virovek, Hayward, CA).

### AAV constructs

The PrP-shRNA sequence was adapted from previous work reported by Dr. Mallucci's group [33], 5′-GTACCGCTACCCTAACCAA-3′, of the mouse mRNA (NM_011170) with a loop sequence, TCTCTTGAA. The constructs of AAV2-PrP-shRNA and AAV2-PrP-shRNA-eGFP were commercially prepared with the U6 promoter in 1×10^13^ vg (vector genome) scale (Virovek, Hayward, CA).

### Animals experiments

Mice were inoculated intrathalamically with 30 µl of 1% brain homogenate containing RML.

#### Drug treatments

Treatments contained 40 mg/kg/day of Qa and 5 mg/kg/day of LY411575 in a chocolate-flavored liquid diet were administrated for 28 days starting at 50 dpi. The chocolate-flavored liquid diet containing Qa and LY411575 was prepared by adding water and cocoa powder (Hershely) to a commercially available liquid mouse diet, LD′82 (Bio-Serv, Inc.). Mice were fed *ad libitum*. Mice were observed daily for signs of neurological disease and euthanized when they were diagnosed with prion disease. Mice were assumed to have prion disease if they exhibited three or more of the following neurological symptoms: aggression, ataxia, circling, dehydration, dysmetria, kyphosis, paralysis, bradykinesia, tremor, tail rigidity, convulsions, depression, weight loss and/or blank stare. Upon diagnosis, mice were euthanized for analysis.

#### Surgical and stereotaxic procedures for intracerebral delivery of AAV2 vector constructs with CED

Mice were weighed and pre-medicated with buprenorphine (Burenex) at 0.07 mg/kg subcutaneous. Induction of anesthesia was done in a 3–5% isofluorane chamber. To prevent drying and irritation of the eyes, sterile ocular lubricant was applied (Puralube Vet Ointment). The mouse was placed into the small animal stereotaxic frame and anesthesia was maintained with a nosecone delivering 1–3% isofluorane. Four µl of AAV2-PrP-shRNA or AAV2-PrP-shRNA-eGFP containing 1×10^13^ vg were bilaterally infused to the thalamus (−1.46 mm posterior to bregma, 2 mm lateral, 3.4 mm ventral) with fused silica capillary tubing (238 µm outer diameter) at 0.5 µl/min. During surgery and recovery, mice were kept on controlled heating pads to maintain normal body temperature and they were then monitored daily for the first 4 days post-surgery.

### BrnAggs culture

BrnAggs were prepared as described previously (Bajsarowicz et al., 2012). Briefly, brain cells from E15 day gestation FVB embryos were dissociated through two nylon meshes. Following two washes with a wash medium (DMEM H21 containing glucose (12 g/L), fungizone (2.5 mg/L) and gentamycin (50 mg/L)), the dissociated cells were resuspended in the growth culture medium (DMEM H21 supplemented with glucose (6 g/L), fungizone and gentamycin and 10% fetal bovine serum (FBS)) at density of 1×10^7^ cells per ml. 4 ml of cells were placed in a 25 ml DeLong flask at constant rotation at 37°C, 10% CO_2_. The next day, 1 ml of exchange medium (DMEM H21 supplemented with glucose, gentamycin and 15% FBS) was added to the flask. After 2–3 days, BrnAggs were transferred to a 50 ml DeLong flask and 5 ml of fresh exchange media were added. BrnAggs were fed every 2–3 days by removing 5 ml of conditioned medium and replacing with 5 ml of fresh exchange medium. At 6–8 days, mature BrnAggs were transferred to a 24-well culture plate, which was rotated constantly. At 15 days in culture they were exposed to a 1 to 50 dilution of RML prions derived from RML-infected CD1 mouse brains. Five days later, BrnAggs were washed with PBS to remove residual RML homogenate; this was followed immediately by a second exposure to RML prions. Uninfected control BrnAggs were prepared the same way but were not exposed to RML prions. After 35–41 days in culture, BrnAggs were harvested and either fixed in 4% paraformaldehyde or sonicated in lysis buffer (10 mM Tris.HCl, pH 8.0, 150 mM NaCl, 0.5% NP40, 5% sodium deoxycholate and protease inhibitors).

### Western blot

5 to 100 µg of lysates or BrnAggs were used for SDS-PAGE gels or protease K (PK) digestion, respectively. For PK digestion, lysates were incubated with PK for 1 hr at 37°C. PMSF (final concentration of 1 mM) was added to stop the digestion. PK-digested samples were centrifuged at 14,000 rpm for 1 hr and then pellets were collected and resuspended with lysis buffer. SDS sample running buffer and reducing reagent (Invitrogen) were added to the samples. Samples were heated to 95°C for 5 min and run in a 4–12% Bis-Tris gel (Invitrogen). The gel was transferred to PVDF membrane with an iBlot system (Invitrogen) and the membrane was blocked with 5% milk for 30 min. The membranes were subsequently incubated overnight with D13-HRP antibody and washed 3 times with TBST for 5 min before developing with ECL reagent (GE).

### Histoblot

Flash frozen brain sections (10 µm) were transferred to the pre-soaked nitrocellulose membrane in histoblot lysis buffer (10 mM Tris-HCl pH 7.8, 100 mM NaCl, 10 mM EDTA, 0.5% NP40, 0.5% sodium deoxycholate) by firmly pressing the slide down onto the membrane for 30 sec. The membrane was air-dried for at least 1 hr, then re-hydrated in TBST for 30 min. For PrP^Sc^ detection of RML-infected brains, the sample was digested with PK (0.2 mg/ml, only for PrP^Sc^ detection) at 37°C for 1 hr. PMSF (5 mM) was added to stop PK digestion. For PrP^C^ detection of uninfected brains, PK-digestion step was replaced by 1 hr incubation in 100 mM NaOH at RT. After PK digestion, the membrane was rinsed with TBST 3 times and then incubated with 10 ml of 3 M guanidine isothiocyanate in Tri/HCl pH 7.8 for 10 min at room temperature (RT), followed by rinse with TBST three times. After blocking with 10 ml of 10% BSA or 5% non-fat milk for 30 min at RT, the membrane was incubated with Hum-P antibody (1∶100) overnight at 4°C. The next day the membrane was rinsed with TBST three times and incubated with an AP conjugated secondary antibody (Promega, Madison, WI; 1∶7500) for 1 hr at RT. The membrane was then rinsed with TBST three times for 10 min and 10 ml of NBT/BCIP (Roche) solution was added for 5 min. After color development, the membrane was washed with DI-H_2_O, PBS, and DI-H_2_O for 1 min each and air-dried. Both treated and untreated groups were transferred and stained in the same membrane to minimize variation in staining. In order to compare the level of PrP^C^ signals, the intergraded densitometry optical measurements of histoblots (6 brain sections per each group) were determined with BioQuant Image software (Nashville, TN).

### Immunofluorescent staining

BrnAggs were washed with PBS for 10 min (2x), fixed in 4% formaldehyde for 3 days at 4°C and washed with PBS for 60 min (3x) and with washing buffer (PBS containing 0.3% Trion-X100 and 0.1% Tween20) for 30 min. For RML-infected BrnAggs, BrnAggs were treated with 3 M guanidine isothiocyanate for 10 min in order to detect PrP^Sc^. For uninfected BrnAggs, this step is skipped and remained in wash buffer. BrnAggs were then washed with wash buffer, blocked with blocking buffer (PBS containing 0.3% Triton-X100, 0.1% Tween20, 2% bovine serum albumin and 10% normal donkey serum) for 3 days at 4°C. Following the incubation with anti-PrP antibodies (D18, R2, and HumP, 1∶250 each) and anti-MAPII antibody (1∶1000), they were washed with wash buffer for 60 min (3x) and incubated with the secondary antibodies, donkey anti-human IgG conjugated with Alexa 594 (1∶800) and donkey anti-rabbit IgG conjugated with Alexa 488 (1∶800) overnight at 4°C. All antibodies were diluted in the blocking buffer. BrnAggs were washed with wash buffer for 60 min (3x), incubated with Hoechst (1∶5000 diluted in PBS) for 30 min at RT and washed with PBS buffer for 30 min (2x). BrnAggs were placed in glycerol containing 2% n-propyl gallate onto a slide and covered with a cover glass. Fluorescent confocal images were taken with the Nikon spectral confocal microscope (Nikon imaging center, UCSF). Dendrite density of BrnAggs was determined by measuring the average MAPII signals of 15 z-stack images of BrnAggs with ImageJ [42].

### Immunohistochemical staining

Mice were perfused with 4% paraformaldehyde and the brains were harvested and fixed in 10% formaldehyde. Following paraffin embedding, sections were prepared in 8- µm-thickness. After deparaffinizing in xylene and hydrating in 95% ethanol, sections were blocked with 3% H_2_O_2_ in methanol for 10 min. After washing, sections were blocked with 10% normal goal serum and incubated with a polyclonal anti-GFP antibody (1∶250, Santa Cruz Biotech, sc-8334) overnight. After washing with PBST (3x), sections were incubated with a goat biotinylated anti-rabbit IgG secondary antibody (1∶100, Vector laboratories, Burlingame, CA) for 1 hr at RT. After incubating with ABC reagents (Vector laboratories), sections were washed and developed with DAB for 1 min (DAB Peroxidase substrate kit, Vector Laboratories). DAB-processed sections were washed in H_2_O and mounted on frosted slides. For hematoxylin eosin (H&E) staining, flash frozen brain sections (10 µm thickness) were fixed for 2 min in 1∶1 methanol (Fisher, A433P) and acetone (Sigma, 179124). The sections were stained with hematoxylin eosin (Fisher Scientific, 245–658) as described previously [43].
